# The Use of Microarray Technology for the Analysis of *Streptococcus Pneumoniae*

**DOI:** 10.1002/cfg.190

**Published:** 2002-08

**Authors:** Jackie McCluskey, Christopher G. Dowson, Timothy J. Mitchell

**Affiliations:** 1 Division of Infection and Immunity Joseph Black Building IBLS University of Glasgow, University Road Glasgow G12 8QQ UK; 2 Department of Biological Sciences University of Warwick Coventry CV4 7AL UK

## Abstract

*Streptococcus pneumoniae* is an important human pathogen associated with pneumonia, septicaemia, meningitis and otitis media. It is estimated to result in over 3 million child deaths worldwide every year and an even greater number of deaths among the elderly. Prior to the complete sequencing of the genomes of *S. pneumoniae* TIGR4 (serotype 4) and *S. pneumoniae* R6 (serotype 2), we designed a custom miniarray consisting of 497 pneumococcal genes. The overall objectives of our microarray investigations were, first, to assess the genetic diversity between different *S. pneumoniae* serotypes, clinical isolates and also different *Streptococcus* species; second, we aimed to use microarray technology to examine the mechanisms by which environmental factors influence pneumococcal gene expression, and ultimately to further the understanding of how these changes in gene expression are achieved and how they may alter the virulence of the organism.


**Comparative and Functional Genomics**
*Comp Funct Genom* 2002; **3**: 366–368.
Published online in Wiley InterScience (www.interscience.wiley.com). **DOI:** 10.1002/cfg.190

**Conference Review**

### The use of microarray technology for the analysis of *Streptococcus pneumoniae*

Jackie McCluskey,[1] Christopher G. Dowson[2] and Timothy J. Mitchell[1]*
[1] *Division of Infection and Immunity, Joseph Black Building, IBLS, University of Glasgow, University Road, Glasgow G12 8QQ, UK*
[2] *Department of Biological Sciences, University of Warwick, Coventry CV4 7AL, UK*

**Correspondence to:*
*Timothy J. Mitchell, Division of Infection and Immunity, Joseph Black Building, IBLS, University of Glasgow, University Road, Glasgow G12 8QQ, UK.*
*E-mail: t.mitchell@bio.gla.ac.uk*

Received: 31 May 2002
Accepted: 12 June 2002

### Abstract

***Streptococcus pneumoniae*** **is an important human pathogen associated with pneumonia, septicaemia, meningitis and otitis media. It is estimated to result in over 3 million child deaths worldwide every year and an even greater number of deaths among the elderly. Prior to the complete sequencing of the genomes of *S. pneumoniae* TIGR4 (serotype 4) and *S. pneumoniae* R6 (serotype 2), we designed a custom miniarray consisting of 497 pneumococcal genes. The overall objectives of our microarray investigations were, first, to assess the genetic diversity between different *S. pneumoniae* serotypes, clinical isolates and also different *Streptococcus* species; second, we aimed to use microarray technology to examine the mechanisms by which environmental factors influence pneumococcal gene expression, and ultimately to further the understanding of how these changes in gene expression are achieved and how they may alter the virulence of the organism. Copyright © 2002 John Wiley & Sons, Ltd.**

**Keywords:** ***Streptococcus pneumoniae*; microarrays; genetic diversity; environmental stimuli; transcriptional profiles**

### Introduction

*Streptococcus pneumoniae* is a Gram-positive coccus belonging to the genus *Streptococcus*, which also includes the oral streptococci *S. oralis* and *S. mitis* [10]. These bacteria live in close proximity in the oral cavity of their animal hosts and the natural transformability of these bacteria has allowed genetic exchange to occur between these different streptococcal species [1]. *S. pneumoniae*, as well as being the major cause of acute bacterial pneumonia and otitis media worldwide, can also exist as a commensal and is known to colonize the upper respiratory tract of up to 40% of humans. To date, as many as 90 distinct capsular serotypes of *S. pneumoniae* have been identified and studies have revealed that certain serotypes are more virulent than others, both in humans and also in a mouse model of infection. Many bacterial factors, including capsule, pneumolysin, autolysin, hyaluronidase and neuraminidase, have been shown to be important for the virulence of the pneumococci [5]. However, a complete understanding of the mechanisms that enable the pneumococci to exist either as a commensal organism, or to cause fatal disease, requires further investigation.

### Comparative genomics of *Streptococcus*

Prior to the complete sequence release and annotation of the genomes of *S. pneumoniae* TIGR4 (serotype 4) by TIGR [9] and *S. pneumoniae* R6 (serotype 2) by Eli Lilly [4] we initiated a project to develop a custom DNA miniarray. This array represented 497 pneumococcal genes. The genes were selected based on their known or putative association with virulence and/or their surface association. Primers were designed using the partial sequences available at the time for *S. pneumoniae* TIGR4 and PCR amplicons for the 497 genes were generated. We initially set out to use our 497 gene miniarray to compare *S. pneumoniae* TIGR4 (serotype 4) with two strains used routinely in our laboratory,

Copyright © 2002 John Wiley & Sons, Ltd.

R6 (non-capsulated serotype 2 derivative) and D39 (serotype 2). Other investigators have since carried out comparative genomic studies of these strains [3,7,9] and the results we obtained with our miniarray were in agreement with these latter studies. We identified a number of genes in TIGR4 which did not hybridize with any in R6 or D39 and these localized to one of the nine gene clusters previously shown to contain differences between these strains [3,9]. These genes included those involved in capsule biosynthesis, an IgA1 protease and putative sortases.

In addition to comparative genomic analysis within the pneumococcus, we also performed preliminary interspecies genomic comparisons. The genetic relationships between different species have been examined previously [3,10]. Our preliminary findings, together with these previous studies, demonstrated that horizontal gene transfer occurs between the species. Whatmore *et al.* [10] had previously demonstrated the occurrence of *ply* and *lytA* in a number of *S. mitis* isolates, which were isolated from disease cases. Analysis of these 'atypical' *S. mitis* isolates using the miniarray demonstrated that in addition to possessing the *lytA* and *ply* genes, they also contained a number of other virulence-associated genes. Further genomic comparisons, both intra- and interspecies, will provide further insight into the frequency of genetic exchange within the genus *Streptococcus*.

### Gene expression analysis of *S. pneumoniae*

The use of microarrays for analysing bacterial gene expression allows exploration of genome-wide expression, as opposed to conventional techniques which would allow analysis of a limited number of transcripts. Using our 497 gene custom miniarray, we aimed to analyse the transcriptional profiles of *S. pneumoniae* grown under different conditions *in vitro* and ultimately examine the expression profiles of organisms isolated from an *in vivo* environment. Our main objective was to further our understanding of the mechanisms used by *Pneumococcus* to alter its gene expression. Previous investigations have utilized microarrays to study the competence regulon of *S. pneumoniae* [8] and also have led to the identification of a novel regulon which is controlled by a small molecular weight peptide [2]. In our investigations, we have constructed a number of mutations in pneumococcal response regulator proteins. We aimed to use the miniarray to compare expression profiles of wild-type and mutant isolates, and ultimately determine which genes might be under the control of these regulator proteins. Preliminary results analysing a *pnpR* mutant [6] demonstrated a number of differences; however, confirmation of these results is still under way.

Construction and use of the pneumococcal miniarray has enabled us to make progress in both the comparative genomics of this organism and expression profile analysis. However, with the release of the complete genomes of *S. pneumoniae* TIGR4 and R6, work is now under way to construct a composite DNA array that will represent the full TIGR4 transcriptome and also additional genes present in R6 but not TIGR4.

### Acknowledgements

We acknowledge BμG@S (the Bacterial Microarray Group at St George's) and The Wellcome Trust for funding their work.

### References

1. Claverys JP, Prudhomme M, Mortier-Barriere I, Martin B. 2000. Adaptation to the environment: *Streptococcus pneumoniae*, a paradigm for recombination-mediated genetic plasticity? *Mol Microbiol* **35**: 251–259.
2. de Saizieu A, Gardes C, Flint N, *et al.* 2000. Microarray-based identification of a novel *Streptococcus pneumoniae* regulon controlled by an autoinduced peptide. *J Bacteriol* **182**: 4696–4703.
3. Hakenbeck R, Balmelle N, Weber B, Gardes C, Keck W, de Saizieu A. 2001. Mosaic genes and mosaic chromosomes: intra- and interspecies genomic variation of *Streptococcus pneumoniae*. *Infect Immun* **69**: 2477–2486.
4. Hoskins J, Alborn WE Jr, Arnold J, *et al.* 2001. Genome of the bacterium *Streptococcus pneumoniae* strain R6. *J Bacteriol* **183**: 5709–5717.
5. Mitchell TJ. 2000. Virulence factors and the pathogenesis of disease caused by *Streptococcus pneumoniae*. *Res Microbiol* **151**: 413–414.
6. Novak R, Cauwels A, Charpentier E, Tuomanen E. 1999. Identification of a *Streptococcus pneumoniae* gene locus encoding proteins of an ABC phosphate transporter and a two-component regulatory system. *Infect Immun* **181**: 1126–1133.
7. Oggioni MR, Pozzi G. 2001. Comparative genomics for identification of clone-specific sequence blocks in *Streptococcus pneumoniae*. *FEMS Microbiol Lett* **200**: 137–143.

Copyright © 2002 John Wiley & Sons, Ltd. 

8. Peterson S, Cline RT, Tettelin H, Sharov V, Morrison DA. 2000. Gene expression analysis of the *Streptococcus pneumoniae* competence regulons by use of DNA microarrays. *J Bacteriol* **182**: 6192–6202.
9. Tettelin H, Nelson KE, Paulsen IT, *et al*. 2001. Complete genome sequence of a virulent isolate of *Streptococcus pneumoniae*. *Science* **293**: 498–506.
10. Whatmore AM, Efstratiou A, Pickerill AP, *et al*. 2000. Genetic relationships between clinical isolates of *Streptococcus pneumoniae*, *Streptococcus oralis* and *Streptococcus mitis*: characterization of 'atypical' pneumococci and organisms allied to *S. mitis* harboring *S. pneumoniae* virulence factor-encoding genes. *Infect Immun* **68**: 1374–1382.

Copyright © 2002 John Wiley & Sons, Ltd. 
